# Genome Sequences of Five Bacteriophages Infecting the Marine *Roseobacter* Bacterium Ruegeria pomeroyi DSS-3

**DOI:** 10.1128/MRA.00959-18

**Published:** 2018-09-27

**Authors:** Yuanchao Zhan, Sijun Huang, Feng Chen

**Affiliations:** a Institute of Marine and Environmental Technology, University of Maryland Center for Environmental Science, Baltimore, Maryland, USA; b CAS Key Laboratory of Tropical Marine Bio-resources and Ecology, South China Sea Institute of Oceanology, Chinese Academy of Sciences, Guangzhou, Guangdong, China; University of California, Riverside

## Abstract

We report the complete genome sequences of five bacteriophages infecting Ruegeria pomeroyi DSS-3, a member of the marine *Roseobacter* lineage. The genomic sequences of these five bacteriophages are almost identical and are closely related to members of the *Chivirus* genus. The genes associated with the lysogenic cycle were also found.

## Abstract (precis)

We report the complete genome sequences of five bacteriophages infecting Ruegeria pomeroyi DSS-3, a member of the marine *Roseobacter* lineage. The genomic sequences of these five bacteriophages are almost identical and are closely related to members of the *Chivirus* genus.

## ANNOUNCEMENT

We report here the genomes of five bacteriophages, DSSΦ1, vB_RpoS-V7, vB_RpoS-V11, vB_RpoS-V16, and vB_RpoS-V18, infecting the marine *Roseobacter* strain Ruegeria pomeroyi DSS-3, which was the first marine *Roseobacter* strain with a published complete genome sequence (1). The five bacteriophages were isolated from the Inner Harbor in Baltimore, MD, using a standard plaque assay (2). The electron microscopy observation indicated that they all belong to the *Siphoviridae*. The genome of DSS3Φ1 was previously sequenced but was not closed (GenBank accession number HQ632855). We resequenced DSS3Φ1 along with four other bacteriophages infecting *R. pomeroyi* DSS-3 on the Illumina MiSeq platform using the Nextera XT DNA library prep kit and the MiSeq reagent kit version 2. More than 1 million reads per library were generated with an average length of 160 bp. The reads with a high rate of ambiguity, low quality, or short length were discarded. CLC Genomic Workbench version 7.5 was used for assembly with the default settings. Finally, 40 to 109 contigs were acquired with *N*_50_ values ranging from 30 kb to 61 kb. Eventually, the complete genomes of five phages were obtained with at least 450× coverage. Genome annotations were done using GeneMarkS version 4.28 and GeneMark.hmm version 3.25 (3) with default settings. tRNA sequences were searched using tRNAscan-SE version 2.0 (4). Predicted protein-coding genes were subjected to a BLAST search (version 2.6.0) against the NCBI nonredundant (NCBI-nr) database, the Conserved Domain Database (CDD), and the Pfam database and then were manually annotated based on the protein product information in GenBank.

The genome size of each of these five roseophages ranges from 59 to 61 kb. The average GC content is 64.0%, nearly identical to that of the host (64.2%). The five bacteriophages contain 82 to 85 predicted genes, only 22 of which were assigned putative functions. Except for vB_RpoS-V16, the genomic sequences of the bacteriophages are almost identical, with 99% nucleotide identities and 99% coverages. The genome of vB_RpoS-V16 shares 97% nucleotide identity over 86% of the genomes of the other four bacteriophages. The major differential regions are located at the end of the genome. The gene encoding the tail tape measure protein of vB_RpoS-V16 is ca. 500 bp shorter than those of the other four phages.

Manual annotation revealed that these five bacteriophages are closely related to the phages within the *Chivirus* genus. *Chivirus* is a genus in the *Siphoviridae* family and contains several phage isolates infecting Escherichia coli (5, 6) and *Salmonella* (7), *Burkholderia* (8), and *Xylella* (9) spp. Members of this genus share an organization similar to that of four major functional modules and have a 15-kb highly divergent left arm of the genome (9). Homolog analysis identified 13 core genes among 5 bacteriophages infecting marine *Roseobacter* spp. and 12 other chi-like phages.

All of these five bacteriophages contain the integrase gene, which is accompanied by a DNA binding protein and a helix-turn-helix domain-containing protein. These genes constitute an integration-related module located between the head morphogenesis and DNA metabolism modules. The finding of this integration module suggests that these bacteriophages may be able to convert to the lysogenic cycle.

### Data availability.

The GenBank accession numbers of the DSSΦ1, vB_RpoS-V7, vB_RpoS-V11, vB_RpoS-V16, and vB_RpoS-V18 genome sequences are KM581061, MH015249, MH015254, MH015258, and MH015252, respectively.
